# SanXoT: a modular and versatile package for the quantitative analysis of high-throughput proteomics experiments

**DOI:** 10.1093/bioinformatics/bty815

**Published:** 2018-09-25

**Authors:** Marco Trevisan-Herraz, Navratan Bagwan, Fernando García-Marqués, Jose Manuel Rodriguez, Inmaculada Jorge, Iakes Ezkurdia, Elena Bonzon-Kulichenko, Jesús Vázquez

**Affiliations:** 1 Vascular Pathophysiology Area, Cardiovascular Proteomics Laboratory, Centro Nacional de Investigaciones Cardiovasculares Carlos III (CNIC), Madrid, Spain; 2 Centro de Investigación Biomédica en Red de Enfermedades Cardiovasculares (CIBERCV), Madrid, Spain

## Abstract

**Summary:**

Mass spectrometry-based proteomics has had a formidable development in recent years, increasing the amount of data handled and the complexity of the statistical resources needed. Here we present SanXoT, an open-source, standalone software package for the statistical analysis of high-throughput, quantitative proteomics experiments. SanXoT is based on our previously developed weighted spectrum, peptide and protein statistical model and has been specifically designed to be modular, scalable and user-configurable. SanXoT allows limitless workflows that adapt to most experimental setups, including quantitative protein analysis in multiple experiments, systems biology, quantification of post-translational modifications and comparison and merging of experimental data from technical or biological replicates.

**Availability and implementation:**

Download links for the SanXoT Software Package, source code and documentation are available at https://wikis.cnic.es/proteomica/index.php/SSP.

**Contact:**

jvazquez@cnic.es or ebonzon@cnic.es

**Supplementary information:**

Supplementary information is available at *Bioinformatics* online.

## 1 Introduction

Current high-throughput quantitative proteomics presents many bioinformatic challenges, especially in the case of stable isotope-based techniques. Several of these problems have been highlighted in the literature, such as the problem of undersampling (Nilsson *et al.*, 2010), the need for a null hypothesis (Arntzen *et al.*, 2011; Karp *et al.*, 2010; Lin *et al.*, 2006), the proteome dynamic range (Zubarev, 2013), the non-normality of protein abundance change distributions (Karp *et al.*, 2010) and the need for quality control measures. Most of these issues were addressed by the weighted spectrum, peptide and protein (WSPP) statistical model (Bonzon-Kulichenko *et al.*, 2011a; García-Marqués *et al.*, 2016; Jorge *et al.*, 2014; Navarro *et al.*, 2014). WSPP models the error structure of the data generated by the mass spectrometer (spectrum level) and integrates the quantitative results into peptide values using weighted averages according to error propagation theory (higher weights are assigned to measurements with lower error). The peptide values are then integrated into protein values and finally the protein values are integrated to determine protein abundance changes. Thus, the data are analysed independently and sequentially at the spectrum, peptide and protein levels and the specific error sources are considered separately, allowing efficient detection of artefacts (Bonzon-Kulichenko *et al.*, 2011a; Bonzon-Kulichenko *et al.*, 2011b; Jorge *et al.*, 2009). The general applicability of WSPP was demonstrated by validating their underlying null hypothesis in a large variety of labelling schemes and mass spectrometers. At each one of the integration levels WSPP uses standardized variables (*z*-values) that have been demonstrated to follow normal distributions in hundreds of experiments (see Refs in Supplementary Material). The characteristics of the WSPP model were firstly exploited in the QuiXoT software package (Trevisan-Herraz *et al.*, 2017). We later generalized the process of data integration according to the WSPP model by developing the Generic Integration Algorithm (GIA), being the Systems Biology Triangle (a method to analyse coordinated protein responses) one of their first applications (García-Marqués *et al.*, 2016). However, we later noticed a growing need for a faster, more flexible, automated and scalable software for protein quantitation, able to cope with large number of experiments and different experimental setups. Hence, we developed the SanXoT software package to fully exploit the robustness, versatility and general applicability of the GIA. SanXoT is very flexible and, thanks to its modularity, can be used in automated workflows. SanXoT has been developed in Python, and is publicly available under the Apache Licence v2.0. It has been extensively tested in Windows, and portable executables for this OS that do not require installation of any further libraries are also available.

## 2 Design

**Fig. 1.**
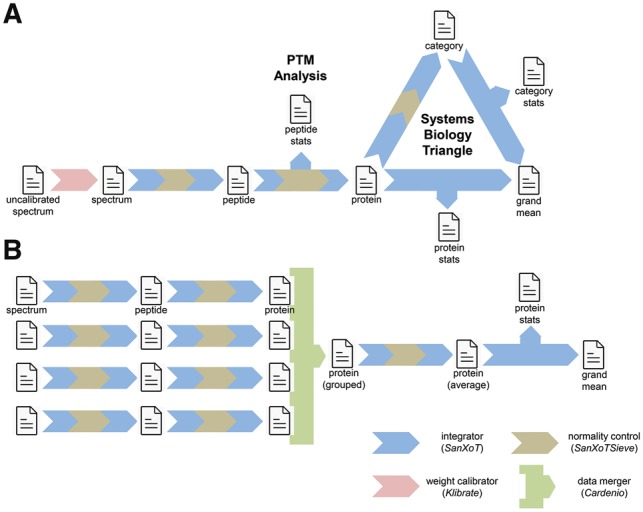
Examples of quantitative workflows constructed with modules from the SanXoT package: (**A**) quantitative analysis of a single experiment, integrating information at the spectrum, peptide and protein levels, including quantitative analysis of post-translational modifications and systems biology analysis using the Systems Biology Triangle; (**B**) quantitative analysis of an experiment performed with four technical or biological replicates. For simplicity, only the four main modules are represented here

SanXoT package workflows follow a modular structure (Fig. 1 and Supplementary Fig. S1), allowing sequential application of the GIA by the SanXoT module*.* A GIA integration consists of integrating the quantitative data from a lower level (such as peptides) into a higher level (such as proteins), as described (García-Marqués *et al.*, 2016). The four main modules of the package are depicted in Figure 1, and details about them are provided in the Supplementary Material.

## 3 Applications

Workflows using prototype versions of SanXoT have been extensively used for systems biology analyses (Fig. 1A) (see Refs in Supplementary Material). They can also be prepared for automated quantification of post-translational modifications (Bagwan *et al.*, 2018), in the global context of protein or functional category changes (Fig. 1A). Workflows can also integrate quantitative data from technical or biological replicates (Fig. 1B). In the latter setup, averaged quantitative values for each protein are calculated taking into account the weight of protein measures in each replicate. The technical or biological variance is automatically calculated in the process. The speed and robustness of SanXoT allows unattended analysis of hundreds of experiments, such as those obtained from clinical cohorts, within one day (manuscript in preparation). A more detailed explanation on these functionalities is available at the Supplementary Material and the wiki at the link provided.

## 4 Conclusion

The successful, extensive use of the SanXoT software package—in preliminary versions—in different biological contexts has demonstrated its utility in exploiting the specific characteristics of the WSPP model for quantitative proteomics analysis (see Refs in Supplementary Material). Perhaps the most notable feature is its robustness, which is mainly a consequence of the use of weighted averages and the estimation of variances from the global distribution of data at each level (Navarro *et al.*, 2014). This was, in turn, possible thanks to the use standardized variables that accurately follow normal distributions in a way unaffected by the undersampling typical of data-independent mass spectrometry approaches.

In addition—thanks to its modular design and the use of plain text files hierarchically structured at each level using relation tables—SanXoT can be easily integrated in other workflows that make use of network analysis or transcriptomics data, or are generated with label-free techniques. SanXoT is currently being adapted for use in parallel with integrated protein identification algorithms, allowing mutual feedback between peptide/protein identification and quantitative information.

## Funding 

This study was supported by competitive grants [BIO2012-37926, BIO2015-67580-P] from the Spanish Ministry of Economy, Industry and Competitiveness (MEIC), [grant IPT13/0001] (ProteoRed, PRB2, ISCIII-SGEFI/ERDF), [grant IPT17/0019] (ProteoRed, PRB3, ISCIII-SGEFI/ERDF), the Fundació La Marató de TV3, and the European Commission FP7 (FP7-PEOPLE-2013-ITN Next generation training in cardiovascular research and innovation-CardioNext). The Centro Nacional de Investigaciones Cardiovasculares Carlos is supported by the Spanish Ministry of Economy, Industry and Competitiveness (MEIC) and the Pro-CNIC Foundation, and is a Severo Ochoa Center of Excellence (MEIC award SEV-2015-0505).

*Conflict of Interest*: none declared.

## Supplementary Material

Supplementary Information
