# Supplementary material for: SanXoT: a modular and versatile package for the quantitative analysis of high-throughput proteomics experiments
Source: Bioinformatics. 2018 Sep 25;35(9):1594–6. doi: 10.1093/bioinformatics/bty815 (PMC6499250; doi:10.1093/bioinformatics/bty815)
Supplement: Supplementary Information [file bty815_supplementary_information.docx]

Supplementary Information for

**SanXoT: a modular and versatile package for the quantitative analysis of high-throughput proteomics experiments**

Marco Trevisan-Herraz^1,2^, Navratan Bagwan^1^, Fernando García-Marqués^1,2^, Jose Manuel Rodriguez^1^, Inmaculada Jorge^1,2^, Iakes Ezkurdia^1,2^, Elena Bonzon-Kulichenko^1,2,*^ and Jesús Vázquez^1,2,*^

^1^ Cardiovascular Proteomics Laboratory, Centro Nacional de Investigaciones Cardiovasculares Carlos III (CNIC), and ^2^ Centro de Investigación Biomédica en Red de Enfermedades Cardiovasculares (CIBERCV), Madrid, Spain

*To whom correspondence should be addressed.

**Contact:** jvazquez@cnic.es; ebonzon@cnic.es

**Documentation and download links:** available at the project’s wiki,

<https://wikis.cnic.es/proteomica/index.php/SSP>

# Main modules

As stated in the main article, the SanXoT software package follows a modular structure, where the four main modules of the package are depicted there in Figure 1. *Klibrate* is used to calibrate statistical weights in the first integration. *SanXoT* integrates lower-level data into higher-level data and calculates the variance of the integration. *SanXoTSieve* performs a normality control on data distributions that are expected to be normal. *Cardenio* helps merging the data from biological or technical replicates (Figure 1B). An additional module, *Aljamia*, aids in the automated generation of tab-separated files needed in some setups, allowing on-the-fly operations and filtering of data.

# Other modules

The SanXoT software package has been designed specially to be applied in a flexible way, so workflows can be adapted to the specific needs of an ongoing research with minimal or no modification of the software. Set-ups include, but are not limited to, systems biology analysis, quantification of post-translational modifications and integration of biological or technical replicates.

**Systems biology:** SanXoT has been applied to a considerable number of systems biology analyses (see references in the *Development and application* section below) in the context of the Systems Biology Triangle (García-Marqués, et al., 2016) in which protein quantifications are integrated into functional categories to detect coordinated protein changes (Figure 1A). In these analyses custom ontological databases can be used to generate the protein-to-category relations files; alternatively, files downloaded using DAVID (Huang, et al., 2009a; Huang, et al., 2009b) can be adapted to produce such protein-to-category relation tables using the module *Camacho*. The module *SanXoTSqueezer* selects the most relevant categories (according to the number of proteins and the category changes), and *Sanson* performs a clustering analysis of categories, allowing the detection of categories redundant in their protein content. This is particularly important when different ontological protein databases are combined. Finally, *SanXoTGauss* generates sigmoid-style graphs highlighting categories that contain proteins changing in a coordinated manner, while *Coordinometer* calculates the *degree of coordination* (García-Marqués, et al., 2016). See Test 3 for a practical example (link in section *Examples*).

**Post-translational modifications:** SanXoT workflows can be prepared for automated quantification of post-translational modifications (PTM) (Bagwan, et al., 2018, submitted), in the global context of protein or functional category changes (Figure 1A). In this setup, *Trilogy* is used to generate peptide-to-protein relations tables in which peptides are tagged as non-modified, modified or orphans (e.g. when the protein is detected only with modified peptides). This allows user-selectable calculation of protein averages and peptide variances using only the unmodified peptide forms. Hence it is possible to detect modified peptides that significantly deviate from the weighted average of the unmodified peptides from the same protein. Protein abundance and systems biology analysis can be performed in the same workflow.

Help and further details for these and additional modules is available at the wiki (link provided above).

# Examples

Users can find in the wiki three unit tests with sample files for a common workflow, including the fundamental workflow (from spectra to peptides, then peptides to proteins and finally quantifying proteins), an integration of two experiments and a systems biology analysis using the Systems Biology Triangle. They are available at:

<https://wikis.cnic.es/proteomica/index.php/Unit_tests_for_SanXoT>

Additionally, a tutorial using the standalone Windows executables including explanations about the details related to the workflows and how they are used in the unit tests is available at:

<https://wikis.cnic.es/proteomica/index.php/Exploring_SanXoT_features>

# Development and application

Preliminary versions of SanXoT **–** and the WSPP statistical model that is behind **–** have been extensively used and tested in recent years for quantitative proteomics experiments. The WSPP has demonstrated to be a robust statistical framework in hundreds of experiments (Baldan-Martin, et al., 2016; Baldanta, et al., 2017; Bartolomé-Izquierdo, et al., 2017; Binek, et al., 2017; Burillo, et al., 2016; Echevarria-Zomeno, et al., 2016; García-Marqués, et al., 2016; Gómez-Serrano, et al., 2016; Gómez-Serrano, et al., 2017; Gonzalez-Calero, et al., 2017; Guaras, et al., 2016; Gulia-Nuss, et al., 2016; Jorge, et al., 2014; Latorre-Pellicer, et al., 2016; Martin-Lorenzo, et al., 2017; Martinez-Acedo, et al., 2012; Navarro, et al., 2014; Quiros, et al., 2014).

The SanXoT software package – in preliminary versions – has successfully put into practice the theoretical background provided by the WSPP statistical method (Bonzon-Kulichenko, et al., 2011; Burillo, et al., 2015; Burillo, et al., 2013; García-Marqués, et al., 2016; Jorge, et al., 2014; Jorge, et al., 2009; Mateos-Hernández, et al., 2016; Navarro, et al., 2014; Perez-Hernandez, et al., 2013; Ramírez-Boo, et al., 2011; Zenón, et al., 2016).

SanXoT is also the cornerstone of the systems biology analysis of a large number of experiments in the context of the Systems Biology Triangle (Baldanta, et al., 2017; Bartolomé-Izquierdo, et al., 2017; Binek, et al., 2017; Burillo, et al., 2016; Caron, et al., 2017; García-Marqués, et al., 2016; Gómez-Serrano, et al., 2017; Martin-Lorenzo, et al., 2017), and novel methods to analyse post-translational modifications (PTM) (Bagwan, et al., 2018, submitted). The final version of SanXoT includes several computational improvements that make it faster and more robust in steps like variance calculation or iterative removal of outliers. These improvements were necessary to reach the degree of stability and automation required to process large amounts of data.

Author contributions

MTH and JV designed SanXoT; MTH developed the software; NB developed the tool for PTM analysis; MTH and JMR implemented and tested the modules; EBK, FGM, IE and IJ benchmarked SanXoT and provided critical evaluation; IE contributed to bioinformatics development; MTH wrote the manuscript. EBK and JV directed the study.

Acknowledgments

We thank Simon Bartlett (CNIC) for English editing. Navratan Bagwan is an FP7-PEOPLE-2013-ITN-CardioNext fellow. The Cardiovascular Proteomics Laboratory is a member of ProteoRed.

References

Bagwan, N.*, et al.* (2018, submitted) Comprehensive quantification of the modified proteome reveals oxidative heart damage in mitochondrial heteroplasmy.

Baldan-Martin, M.*, et al.* (2016) Plasma Molecular Signatures in Hypertensive Patients With Renin-Angiotensin System Suppression: New Predictors of Renal Damage and De Novo Albuminuria Indicators. *Hypertension*, 68, 157-166.

Baldanta, S.*, et al.* (2017) ISG15 governs mitochondrial function in macrophages following vaccinia virus infection. *PLoS Pathog.*, 13, e1006651.

Bartolomé-Izquierdo, N.*, et al.* (2017) miR-28 regulates the germinal center reaction and blocks tumor growth in preclinical models of non-Hodgkin lymphoma. *Blood*, 129, 2408-2419.

Binek, A.*, et al.* (2017) Proteomic footprint of myocardial ischemia/reperfusion injury: Longitudinal study of the at-risk and remote regions in the pig model. *Sci. Rep.*, 7, 12343.

Bonzon-Kulichenko, E.*, et al.* (2011) Quantitative in-depth analysis of the dynamic secretome of activated Jurkat T-cells. *J. Proteomics*, 75, 561-571.

Burillo, E.*, et al.* (2016) Quantitative HDL Proteomics Identifies Peroxiredoxin-6 as a Biomarker of Human Abdominal Aortic Aneurysm. *Sci. Rep.*, 6, 38477.

Burillo, E.*, et al.* (2015) ApoA-I/HDL-C levels are inversely associated with abdominal aortic aneurysm progression. *Thromb. Haemost.*, 113, 1335-1346.

Burillo, E., Vazquez, J. and Jorge, I. (2013) Quantitative proteomics analysis of high-density lipoproteins by stable 18O-isotope labeling. *Methods Mol. Biol.*, 1000, 139-156.

Caron, E.*, et al.* (2017) Precise Temporal Profiling of Signaling Complexes in Primary Cells Using SWATH Mass Spectrometry. *Cell Rep.*, 18, 3219-3226.

Echevarria-Zomeno, S.*, et al.* (2016) Dissecting the proteome dynamics of the early heat stress response leading to plant survival or death in Arabidopsis. *Plant Cell Environ*, 39, 1264-1278.

García-Marqués, F.*, et al.* (2016) A Novel Systems-Biology Algorithm for the Analysis of Coordinated Protein Responses Using Quantitative Proteomics. *Mol. Cell. Proteomics*, 15, 1740-1760.

Gómez-Serrano, M.*, et al.* (2016) Proteome-wide alterations on adipose tissue from obese patients as age-, diabetes-and gender-specific hallmarks. *Scientific reports*, 6, 25756.

Gómez-Serrano, M.*, et al.* (2017) Differential proteomic and oxidative profiles unveil dysfunctional protein import to adipocyte mitochondria in obesity-associated aging and diabetes. *Redox Biol*, 11, 415-428.

Gonzalez-Calero, L.*, et al.* (2017) Urinary exosomes reveal protein signatures in hypertensive patients with albuminuria. *Oncotarget*, 8, 44217-44231.

Guaras, A.*, et al.* (2016) The CoQH2/CoQ Ratio Serves as a Sensor of Respiratory Chain Efficiency. *Cell Rep*, 15, 197-209.

Gulia-Nuss, M.*, et al.* (2016) Genomic insights into the Ixodes scapularis tick vector of Lyme disease. *Nat Commun*, 7, 10507.

Huang, D.W., Sherman, B.T. and Lempicki, R.A. (2009a) Bioinformatics enrichment tools: paths toward the comprehensive functional analysis of large gene lists. *Nucleic Acids Res.*, 37, 1-13.ç

Huang, D.W., Sherman, B.T. and Lempicki, R.A. (2009b) Systematic and integrative analysis of large gene lists using DAVID bioinformatics resources. *Nat. Protoc.*, 4, 44-57.

Jorge, I.*, et al.* (2014) The human HDL proteome displays high inter-individual variability and is altered dynamically in response to angioplasty-induced atheroma plaque rupture. *J. Proteomics*, 106, 61-73.

Jorge, I.*, et al.* (2009) Statistical model to analyze quantitative proteomics data obtained by 18O/16O labeling and linear ion trap mass spectrometry: application to the study of vascular endothelial growth factor-induced angiogenesis in endothelial cells. *Mol. Cell. Proteomics*, 8, 1130-1149.

Latorre-Pellicer, A.*, et al.* (2016) Mitochondrial and nuclear DNA matching shapes metabolism and healthy ageing. *Nature*, 535, 561-565.

Martin-Lorenzo, M.*, et al.* (2017) Immune system deregulation in hypertensive patients chronically RAS suppressed developing albuminuria. *Sci. Rep.*, 7, 8894.

Martinez-Acedo, P.*, et al.* (2012) A novel strategy for global analysis of the dynamic thiol redox proteome. *Molecular & cellular proteomics : MCP*, 11, 800-813.

Mateos-Hernández, L.*, et al.* (2016) Quantitative proteomics reveals Piccolo as a candidate serological correlate of recovery from Guillain-Barré syndrome. *Oncotarget*, 7, 74582-74591.

Navarro, P.*, et al.* (2014) General statistical framework for quantitative proteomics by stable isotope labeling. *J. Proteome Res.*, 13, 1234-1247.

Perez-Hernandez, D.*, et al.* (2013) The intracellular interactome of tetraspanin-enriched microdomains reveals their function as sorting machineries toward exosomes. *J. Biol. Chem.*, 288, 11649-11661.

Quiros, P.M.*, et al.* (2014) ATP-dependent Lon protease controls tumor bioenergetics by reprogramming mitochondrial activity. *Cell Rep*, 8, 542-556.

Ramírez-Boo, M.*, et al.* (2011) Quantitative proteomics by 2-DE, 16O/18O labelling and linear ion trap mass spectrometry analysis of lymph nodes from piglets inoculated by porcine circovirus type 2. *Proteomics*, 11, 3452-3469.

Zenón, F.*, et al.* (2016) 18O proteomics reveal increased human apolipoprotein CIII in Hispanic HIV-1+ women with HAART that use cocaine. *Proteomics Clin. Appl.*, 10, 144-155.
